# Standard of care for children and adolescents with chronic kidney disease and its Peculiarities: a call to action!

**DOI:** 10.1590/2175-8239-JBN-2025-0178en

**Published:** 2025-12-19

**Authors:** Lilian Monteiro Pereira Palma, Maria Goretti Moreira Guimarães Penido, Emília Maria Dantas Soeiro, Marcelo de Sousa Tavares, Clotilde Druck Garcia, Arnauld Kaufman, Suzana Aparecida Greggi de Alcantara, Olberes Vitor Braga de Andrade, Káthia Liliane da Cunha Ribeiro Zuntini, Roberta Mendes Lima Sobral, Vandrea Carla de Souza, Rejane de Paula Bernardes, Maria Cristina de Andrade, Nilzete Liberato Bresolin, Paulo Cesar Koch Nogueira, Vera Hermina Kalika Koch

**Affiliations:** 1 Universidade Estadual de Campinas, Hospital de Clínicas, Departamento de Pediatria, Nefrologia Pediátrica, Campinas, SP, Brazil.; 2 Santa Casa de Belo Horizonte, Unidade de Nefrologia Pediátrica, Belo Horizonte, MG, Brazil.; 3 Universidade Federal de Pernambuco, Área Acadêmica de Pediatria Recife, PE, Brazil.; 4 Instituto de Medicina Integral Professor Fernando Figueira (IMIP), Unidade Renal Pediátrica, Recife, PE, Brazil.; 5 Universidade Federal Ciências da Saúde do Rio Grande do Sul, RS, Brazil.; 6 Santa Casa de Porto Alegre, Transplante Pediátrico, Porto Alegre, RS, Brazil.; 7 Universidade Federal do Rio de Janeiro, Rio de Janeiro, RJ, Brazil.; 8 Universidade de São Paulo, Hospital das Clínicas da Faculdade de Medicina de Ribeirão Preto, Nefrologia Pediatrica, SP, Brazil.; 9 Santa Casa de Misericórdia de São Paulo, Faculdade de Ciências Médicas, Departamento de Pediatria, São Paulo, SP, Brazil.; 10 Hospital Infantil Albert Sabin, Fortaleza, CE, Brazil.; 11 Universidade Federal da Bahia, Hospital Universitário Prof. Edgard Santos, Nefrologia Pediátrica, Salvador, BA, Brazil.; 12 Universidade de Caxias do Sul, Programa de Pós Graduação em Ciências da Saúde, Caxias do Sul, RS, Brazil.; 13 Instituição: Clínica NefroKids, Curitiba, PR, Brazil.; 14 Universidade Federal de São Paulo, Escola Paulista de Medicina, Nefrologia Pediátrica – São Paulo, SP, Brazil.; 15 Universidade Federal de Santa Catarina, Hospital Joana de Gusmão, Nefrologia Pediátrica, Florianópolis, SC, Brazil.; 16 Hospital Samaritano, São Paulo, SP, Brazil.; 17 Universidade de São Paulo, SP, Brazil.

**Keywords:** Standard of Care, Renal Insufficiency, Chronic, Pediatric Nephrology, Child, Adolescent

## Abstract

The Pediatric Nephrology Standard of Care is essential for the diagnosis, monitoring, and management of children with Chronic Kidney Disease (CKD). Early detection of kidney changes is essential since many conditions can be silent, but have a major impact on the child's growth and development. The Standard of Care structure includes neonatal screening, assessment of predisposing conditions for kidney disease, calculation of the estimate of renal function and classification of the CKD stage to propose drug and nondrug therapeutic interventions. Challenges such as shortage of specialists, need for screenings and unequal access to services reinforce the importance of robust public policies and training programs. Personalized protocols are recommended to delay progression to Renal Replacement Therapy. In September 2024, the Clinical Guidelines and Therapeutic Protocol (CGTP) for CKD in adults was launched. In this Opinion article, a group of Pediatric Nephrologists comment on the perspectives for creating a CGTP for children and adolescents with CKD.

## Introduction

On September 16, 2024, the Clinical Guidelines and Therapeutic Protocol (CGTP) for Strategies to Slow the Progression of Chronic Kidney Disease (CKD)^1^ in adults was approved. The document considers the growing prevalence of CKD in Brazil (9% of the population)^2^ and its impact on public health, given the high maintenance costs of patients undergoing all forms of Renal Replacement Therapy (RRT): hemodialysis, peritoneal dialysis, and kidney transplantation.

Studies show that implementing a “Standard of Care in Nephrology” improves the quality of life of CKD patients, slows the progression of kidney disease, and delays the need for dialysis and kidney transplantation, resulting in savings in both financial^3^ and natural resources.

Screening for CKD in at-risk groups is essential. Primary Health Care (PHC) is responsible for recognizing conditions associated with the potential progression of kidney disease. For adults, better control of systemic arterial hypertension (SAH) and *diabetes mellitus*, early detection of CKD using formulas to calculate the glomerular filtration rate (GFR), urine and imaging tests, and guidance on lifestyle changes are recommended. Regarding adults, the therapeutic arsenal proposed in the CGTP^1^ includes angiotensin-converting enzyme (ACE) inhibitors, angiotensin receptor blockers (ARBs), aldosterone antagonists, sodium-glucose cotransporter 2 inhibitors (SGLT2 inhibitors), and the conservative management of anemia, mineral and bone disorder, SAH, acidemia, and cardiovascular disease. However, these guidelines do not cover the specific needs of the pediatric population.

### What are the Needs of the Pediatric Population?

Pediatric Nephrology is the area of Pediatrics responsible for diagnosing, monitoring, and treating conditions that may affect the kidneys of children and adolescents. The most common causes of nephropathy in this age group differ from those observed in the adult population and include urinary tract malformations, glomerulopathies, genetic kidney diseases, tubulopathies, and acquired conditions (kidney damage secondary to sepsis and other infectious diseases, use of nephrotoxic drugs and chemotherapy). Since conception, an unfavorable intrauterine environment may promote adaptations (epigenetic phenomena) that impair renal maturation^4^. This process is referred to as Fetal and Postnatal Programming of Adult Kidney Disease.

Technological advances have enabled the survival of preterm infants, low birth weight newborns^5^, children undergoing complex surgeries, those with cancer who have received nephrotoxic chemotherapy, and children admitted to Intensive Care Units. Under these conditions, there is potential for a slow and gradual loss of renal function over the years, and initial recognition and intervention fall to the PHC team.

**Figure 1:**
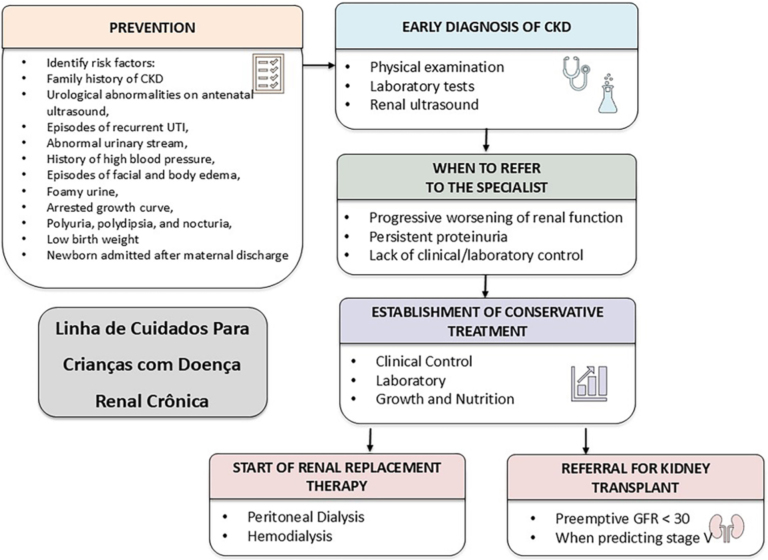
General framework of the standard of care in pediatric nephrology.

Another important factor is the increase in pediatric obesity. As a consequence, SAH is becoming increasingly prevalent in this population and is associated with worsening kidney function when left untreated. Studies have shown that public intervention programs against childhood obesity in schools are not effective if they fail to consider other sources of support for a healthy lifestyle, especially those provided by the family^6^ (healthy eating, combating sedentary lifestyles, encouraging physical activity, and sleep hygiene). To be successful, a comprehensive approach to the patient and their family is required. A solely medical approach is not sufficient to achieve more effective therapy, not only at the PHC level, but especially at the secondary and tertiary levels. These data justify the creation of a Pediatric Nephrology Standard of Care (Figure 1).

### How to Prevent CKD in Children?

#### Caring for the expectant mother

- Avoid the use of ACE inhibitors, ARBs, nonsteroidal antiinflammatory drugs (NSAIDs), illicit drugs, tobacco, and alcohol
- Prevent and treat overweight/obesity, metabolic syndrome, and gestational diabetes
- Diagnose and treat dyslipidemias
- Diagnose and treat hypertension
- Control infectious diseases (rubella, toxoplasmosis, cytomegalovirus, etc.)
- Perform genetic counseling
- Encourage the use of folic acid
- Ensure proper nutrition for the expectant mother
- Recognize and diagnose genetic diseases with potential transmission to the fetus
- Ensure adequate prenatal care, with high-quality fetal ultrasound, to identify urinary tract malformations and spinal dysraphisms
- Refer pregnant women to tertiary care hospitals (neonatology and pediatric nephrology) when a urinary tract malformation is diagnosed

To achieve these goals, coordinated action with gynecologists and obstetricians is required, as well as primary intervention with the multidisciplinary team at Primary Health Care Centers (UBSs in Portuguese).

#### Caring for the Newborn (NB):

- Avoid the use of ACE inhibitors, ARBs, NSAIDs, as well as the mother’s use of illicit drugs, tobacco, and alcohol
- Implement educational measures with the neonatology team for monitoring NBs at risk of CKD progression
- Emphasize that acute kidney injury (AKI) is an important risk factor for progression to CKD (these infants require follow-up after discharge, including assessment of kidney function, detection of proteinuria/microalbuminuria, and monitoring of blood pressure levels)
- Minimize exposure to nephrotoxic agents (aminoglycosides, non-hormonal antiinflammatory drugs)
- Highlight the importance of serum creatinine and disseminate reference tables by age group, in addition to the formula for calculating GFR
- Avoid the use of ACE inhibitors (especially before 44 weeks of corrected gestational age), ARBs, NSAIDs, as well as the mother’s use of illicit drugs, tobacco, and alcohol
- Encourage breastfeeding
- Measure blood pressure in at-risk groups
- Ensure adequate nutrition for the infant
- Assess postnatal growth catch-up (recovery)
- Refer AKI patients for follow-up with a pediatric nephrologist
- Explain the importance of serum creatinine and disseminate reference tables by age group, in addition to the formula for calculating GFR

To achieve these goals, joint action among pediatricians, parents/caregivers, and the multidisciplinary team at the UBSs is required.

#### Caring for children and adolescents

- Provide guidance on complementary feeding
- Promoting nutrition education
- Measure blood pressure annually in eutrophic children starting at three years of age
- Frequently measure blood pressure in patients under three years of age among at-risk groups
- Encourage regular physical activity under professional supervision
- Prevent overweight/obesity and dyslipidemias
- Provide guidance on the harmful effects of illicit drugs, tobacco, and alcohol use
- Explain the importance of serum creatinine and disseminate reference tables by age group, in addition to the formula for calculating GFR
- At all ages, provide guidance on assessing anthropometric data, measuring serum creatinine to calculate GFR, and checking blood pressure, as these measurements serve as warning signs for chronic diseases. In addition, provide guidance on the importance of adequate immunization schedule across all age groups

The fundamental role of the nursing team, nutritionists, social workers, and mental health team in providing psychological and emotional support across all care lines involved in this process should be emphasized.

### What are the Particularities of Diagnosing Kidney Disease in the Pediatric Population? How do they Differ from those in Adults?

The diagnosis of pediatric CKD begins with the evaluation of the child’s family and perinatal history, complemented by fetal ultrasonography, a fundamental diagnostic tool for detecting urinary system malformations, as well as morphological manifestations of genetically determined diseases.

Kidney disease is often asymptomatic; however, it may present with signs and symptoms such as abnormal urinalysis, urinary tract infection, electrolyte and acid-base disorders, congenital abnormalities of the kidney and urinary tract, renal involvement in systemic disease, glomerular disease, renal tubular disorder, and SAH. Therefore, a complete medical history and physical examination are essential for diagnosis. The physical examination must include blood pressure measurement, and family and personal history should always be investigated. Pediatric and adult kidney diseases differ regarding causes, clinical presentation, and long-term consequences (progressive renal effects, growth, and neurocognitive development). It is essential to recognize that, although the basic principles of diagnosis and treatment are similar, the impact of kidney disease on growth and development requires age-specific considerations for children and adolescents^7^.

### Challenges in Estimating GFR in the Pediatric Population

The difficulty in defining pediatric CKD using GFR is demonstrated by the KDIGO (Kidney Disease Improving Global Outcomes) definition, which, in its latest edition^8,9^, establishes the lower limit of normal for estimated GFR (eGFR) – calculated from serum creatinine levels – as ≥90 mL/min/1.73 m^2^ for children and adolescents aged >2 years. Between 1 and 2 years of age, a child’s GFR already reaches levels comparable to those of an adult.

### How does GFR vary in Children Under 2 Years of Age?

Nephrogenesis is completed between the 32nd and 36th weeks of gestation. It is estimated that for each additional kilogram of weight, approximately 250,000 nephrons are formed per kidney. By the end of gestation, this process culminates in about one million nephrons in each kidney of a full-term newborn^13^. In term neonates, postnatal nephrogenesis does not occur. In preterm infants, however, the process may persist for up to approximately 40 days after birth, usually ceasing two weeks before the expected corrected age. In this extrauterine environment, the nephrons formed are more vulnerable to malformations and dysfunctions, underscoring the importance of renal protection strategies in the neonatal period^10,11,12^. A significant portion of nephron formation (60%) occurs in the third trimester of pregnancy; therefore, the more extreme the prematurity, the lower the total number of nephrons at birth^14^. The GFR of neonates correlates with gestational age at birth and is lower in preterm infants than in term NBs. In the first days of life, neonates have serum creatinine levels similar to those of their mothers, followed by a progressive increase in GFR, according to the number of functioning nephrons and muscle mass^15^ (Table 1).

**Table 1:** Glomerular filtration rate (inulin clearance) in the pediatric population aged < 2 years^16^

Age	Mean GFR ± SD mL/min/1.73 m^2^ BSA
Preterm NB 1–7 days	18.7 ± 5.5
Preterm NB aged 1.5–4 months	67.4 ± 16.6
Term NB 1–3 days	20.8 ± 5.0
Term NB 1–3 months	85.3 ± 35.1
Term NB 7–12 months	96.2 ± 12.2
Term NB 1–2 years old	105.2 ± 17.3

Abbreviation – GFR: glomerular filtration rate; SD: standard deviation; BSA: body surface area; NB: newborn.

GFR assessment in children aged one year or older and in adolescents is performed using formulas and is referred to as estimated GFR (eGFR)^17^. The recommended equation for this assessment is the CKiDU-25, based on serum or plasma creatinine, or on cystatin C^18^. This equation may be calculated online at **https://ckid-gfrcalculator.shinyapps.io/eGFR/**. The 2024 KDIGO guidelines^9^ recommend that the eGFR calculation for children and young adults with altered muscle mass be determined based on cystatin C (e.g., in cases of malformations and lower limb atrophy, spinal dysraphism, and muscle diseases).

### When does Conservative CKD Treatment Begin?

Conservative treatment should be initiated after the diagnosis of CKD, while the disease is still in its early stages, in order to increase the chances of preserving kidney function and delaying the need for RRT. Certain signs and symptoms are a warning indicator of the likelihood of the disease, contributing to early diagnosis^19,20^. These include:

1) Signs related to urinary tract malformations (urological abnormalities on antenatal ultrasonography, recurrent UTIs, abnormal urinary stream);
2) Signs of glomerular disease (history of SAH, facial and body edema, foamy urine);
3) Flattening of the growth curve;
4) Signs of impaired urine concentration ability (polyuria, polydipsia, nocturia);
5) Low birth weight;
6) History of hospitalization during the neonatal period^20^ (especially when the mother is discharged but the NB remains hospitalized).

#### Pillars of conservative treatment

**Figure 2:**
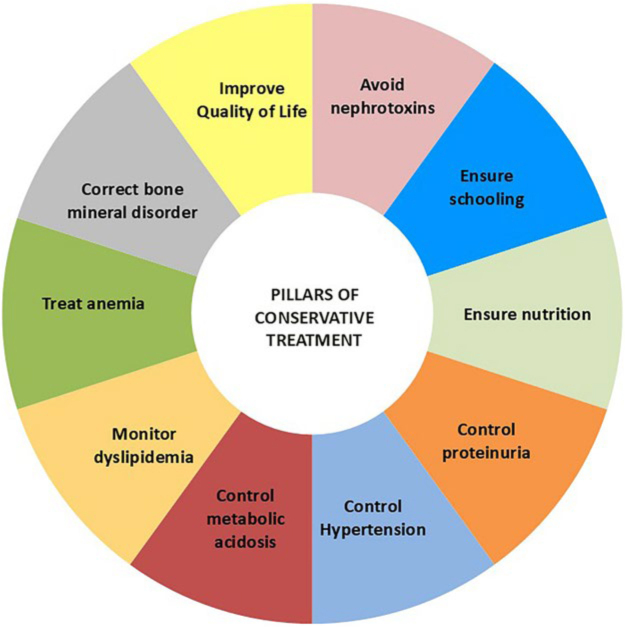
Pillars of conservative treatment of chronic kidney disease in the pediatric population.

- Preservation of kidney function through regular monitoring of GFR, management of hydration and mineral disorders, while avoiding nephrotoxins
- Growth monitoring and treatment of short stature
- Lifestyle changes: age-appropriate physical activity, healthy diet, and reduced sodium intake, while avoiding protein restriction due to the risk of impaired growth
- Symptom management (nausea, loss of appetite, and illness-related emotional distress)
- Early detection of albuminuria and proteinuria, with control through drug and non-drug therapeutic measures
- Management of SAH, anemia, mineral and bone disorders, and other comorbidities
- Maintaining quality of life (school absenteeism with significant damage to cognitive and social development)
- Encouragement of family and patient participation in decision-making regarding all aspects of the child’s treatment (Figure 2).

### RRT – Dialysis and Kidney Transplantation – What are the Needs?

In 2015, a study showed that in Brazil, the number of children undergoing dialysis or transplantation reached 1,283, with a prevalence of 20 cases per million population (pmp) and an incidence of 6.6 pmp. Regional disparities were observed across the country, both in terms of population profile and the type and quality of treatment provided^21^.

Peritoneal dialysis (PD) is a high-quality, lowcost modality of artificial renal support and is considered the therapy of choice for children and adolescents. Its clinical outcomes, as well as those related to quality of life, are comparable—if not superior—to those obtained with hemodialysis (HD). More than 50% of pediatric patients with end-stage CKD initiate RRT through PD. The goal of this therapy is to prepare the patient for a timely kidney transplantation.

The advantages of PD compared to HD include: preservation of residual renal function, fewer dietary restrictions, possibility of school attendance, no need for vascular access, lower risk of potential growth impairment, preservation of vascular access, and maintenance of social interaction. Several studies have indicated this therapy as the best option for infants and young children^22^.

The lack of PD provision for pediatric patients prevents the survival of many who do not meet the minimum weight requirement for HD. Newborns and infants only a few months old, as well as those with low birth weight, extreme prematurity, or birth conditions, have PD as their only chance of survival until a possible kidney transplant.

A Brazilian study published in 2024 demonstrated the regional inequality in travel distances to dialysis units across the country^23^. In the South and Southeast regions, where there is a higher density of facilities, patients have greater local availability of centers, traveling shorter distances (median of 27.6 km to the dialysis unit). In contrast, in the North region,this distance is significantly greater (median of 84.3 km), and 77% of patients need to travel more than 40 km to receive treatment. On February 19, 2024, the *Agência Câmara de Notícias* announced a bill establishing that hemodialysis units must be located within 100 km of the patient’s residence. This is Bill No. 6133/23 (camara.leg.br), which requires public administration to ensure the availability of HD units within a radius of up to 100 km from patients. The bill also provides for oversight and monitoring mechanisms to ensure compliance with distance limits and quality of care in HD units. As of the publication of this article, the proposal was still awaiting review by the Health, Finance, and Constitution and Justice Committees of the Brazilian Chamber of Deputies.

The Brazilian Pediatric Nephrology community has grown and is now present in all Brazilian states and the Federal District. However, the number of services with pediatric nephrologists remains insufficient to meet the demand of patients requiring RRT (data from the Pediatric Nephrology Department of the Brazilian Society of Nephrology). Although pediatric nephrologists are trained and qualified to manage a wide range of kidney diseases and their complications, not all services have the necessary support to initiate dialysis in children and newborns. There is significant regional disparity, and in some situations, patients are unable to wait for transfer to a specialized dialysis center, resulting in death at the referring facility.

In 2022, a Brazilian cross-sectional study was conducted to identify the number of RRT centers in the country that use PD in pediatrics^24^. The study showed that 212 pediatric patients were enrolled in PD programs across 37 centers, most of whom were children aged 0 to 12 years. Automated PD was the primary modality used (86%), and in 74% of cases, the payer was the Brazilian Unified Health System (SUS). In this study, Vantive^®^ (formerly Baxter^®^) was identified as the main supplier of machines and inputs for pediatric PD in Brazil, holding more than 80% of the market. However, around 25% of services reported experiencing shortages of supplies in the preceding months, resulting in the conversion of pediatric patients from PD to HD in 19 cases (51%).

For the vast majority of children with CKD, kidney transplantation is the treatment of choice. However, a mapping conducted in 2015 revealed an uneven distribution in the frequency of transplants among Brazil’s regions^25^. The lowest rate was recorded in the Central-West region, with 0.4 age-related cases per million population (pmp), whereas the highest was observed in the South region, with 8.3 cases pmp. Children from the North and Central-West regions had a 3–4-fold lower likelihood of receiving a deceased donor transplant (p < 0.05). In addition to geographic region, recipient age and local development index also influenced the results. The probability of receiving a kidney transplant was very low among young children in those regions. The authors recommend referring these children to a Kidney Transplant Center when GFR is <30 mL/min/1.73 m^2^, which allows for careful evaluation and follow-up, as well as preparation of the lower urinary tract, when necessary, aiming to increase the possibility of performing a preemptive kidney transplant.

### What Strategies are Necessary to Establish a Standard of Care in Pediatrics?

Unlike what occurs in adults, there is still no systematized Standard of Care in Pediatric Nephrology. The fact that most clinical studies exclude pediatric patients, particularly those under 12 years of age, results in the off-label use of a large proportion of medications. In addition, reduced food intake and dietary restrictions in the more advanced stages of CKD impair the child’s growth and development. The requirements currently used by pharmacies for dispensing high-cost drugs do not address pediatric needs. These issues alone justify a specific algorithm for pediatric CKD care, involving a multidisciplinary team composed of psychologists, nutritionists, nurses, and occupational therapists.

- Develop a bipartisan guideline, with members of the Pediatric Nephrology Departments of the Brazilian Societies of Nephrology and of Pediatrics, with the creation of a pediatric-specific algorithm, similar to the CGTP for adults, published on September 16, 2024^1^;
- Include pharmacological treatment and the promotion of healthy lifestyle habits, bearing in mind that the fundamental pillar is the family’s support and engagement;
- Provide nutritional guidance on CKD, with practical and low-cost menu options suitable for each disease stage, according to age group;
- Study the release of drugs with proven costeffectiveness in clinical trials and already approved in international markets for adult use, following regulatory guidelines. Examples include sevalamer, cinacalcet, SGLT2 inhibitors, and sodium zirconium cyclosilicate;
- While awaiting approval for pediatric use of certain drugs, consider off-label use of those with proven efficacy and cost-effectiveness in the adult population, such as SLGT2 inhibitors (gliflozins);
- Make the creation of multidisciplinary teams a reality, focused on a comprehensive approach to the care of children with CKD and their families;
- Refer CKD patients to Pediatric Transplant Centers at an early stage;
- Plan the transition of care to adult nephrology based on initiatives that promote patient autonomy^26,27^.

## Conclusion

Defining a Standard of Care in Pediatric Nephrology and engaging the family in the treatment are fundamental steps to delay the onset of RRT. Promoting rapid access to kidney transplant centers and training new services is a national emergency.

Let’s take action!

## Data Availability

No new data were generated or analyzed in this study.

## References

[1] Brasil, Ministério da Saúde. Portaria Conjunta SAES/SECTICS nº 11,
de 16 de setembro de 2024 Protocolo Clínico e Diretrizes Terapêuticas das Estratégias para
Atenuar a Progressão da Doença Renal Crônica. Diário Oficial da União [Internet]; Brasília.

[2] Barreto SM, Ladeira ML, Duncan BB, Schmidt MI, Lopes AA, Benseñor IM (2016). Chronic kidney disease among adult participants of the
ELSA-Brasil cohort: association with race and socioeconomic
position. J Epidemiol Community Health.

[3] Moraes CS, Fernandes NMDS, Colugnati FAB (2021). Multidisciplinary treatment for patients with chronic kidney
disease in pre-dialysis minimizes costs: a four-year restrospective cohort
analysis. J Bras Nefrol.

[4] Minuth WW (2020). Shaping of the nephron: a complex, vulnerable, and poorly
explored backdrop for noxae impairing nephrogenesis in the fetal human
kidney. Mol Cell Pediatr.

[5] Andrade MC, Bresolin NL, Brecheret AP (2024). Low birth weight and renal consequences: knowing about it means
preventing it. J Bras Nefrol.

[6] Adab P, Pallan MJ, Lancashire ER, Hemming K, Frew E, Barrett T (2018). Effectiveness of a childhood obesity prevention programme
delivered through schools, targeting 6 and 7 year olds: cluster randomised
controlled trial (WAVES study). BMJ.

[7] Barakat AJ (2012). Presentation of the child with renal disease and guidelines for
referral to the pediatric nephrologist. Int J Pediatr.

[8] Cirillo L, De Chiara L, Innocenti S, Errichiello C, Romagnani P, Becherucci F (2023). Chronic kidney disease in children: an update. Clin Kidney J.

[9] Stevens PE, Ahmed SB, Carrero JJ, Foster B, Francis A, Hall RK (2024). Kidney Disease: Improving Global Outcomes (KDIGO) CKD Work Group.
KDIGO 2024 Clinical Practice guideline for the evaluation and management of
chronic kidney disease. Kidney Int.

[10] Carpenter J, Yarlagadda S, VandenHeuvel KA, Ding L, Schuh MP (2023). Human nephrogenesis can persist beyond 40 postnatal days in
preterm infants. Kidney Int Rep.

[11] Sutherland MR, Black MJ (2023). The impact of intrauterine growth restriction and prematurity on
nephron endowment. Nat Rev Nephrol.

[12] Rodríguez MM, Gomez AH, Abitbol CL, Chandar JJ, Duara S, Zilleruelo GE (2004). Histomorphometric analysis of postnatal glomerulogenesis in
extremely preterm infants. Pediatr Dev Pathol.

[13] Hughson M, Farris 3rd, Douglas-Denton R, Hoy WE, Bertram JF (2003). Glomerular number and size in autopsy kidneys: the relationship
to birth weight. Kidney Int.

[14] Mañalich R, Reyes L, Herrera M, Melendi C, Fundora I (2000). Relationship between weight at birth and the number and size of
renal glomeruli in humans: a histomorphometric study. Kidney Int.

[15] Sulemanji M, Vakili K (2013). Neonatal renal physiology. Semin Pediatr Surg.

[16] Schwartz GJ, Furth SL (2007). Glomerular filtration rate measurement and estimation in chronic
kidney disease. Pediatr Nephrol.

[17] Bacchetta J, Cochat P, Rognant N, Ranchin B, Hadj-Aissa A, Dubourg L (2011). Which creatinine and cystatin C equations can be reliably used in
children?. Clin J Am Soc Nephrol.

[18] Pierce CB, Muñoz A, Ng DK, Warady BA, Furth SL, Schwartz GJ (2021). Age- and sex-dependent clinical equations to estimate glomerular
filtration rates in children and young adults with chronic kidney
disease. Kidney Int.

[19] Koch-Nogueira PC, Feltran LS, Camargo MF, Leão ER, Benninghoven CS, Gonçalves NZ (2011). Prevalência estimada da doença renal crônica terminal em crianças
no Estado de São Paulo. Rev Assoc Med Bras.

[20] Koch-Nogueira PC, Venson AH, Carvalho MFC, Konstantyner T, Sesso R (2023). Symptoms for early diagnosis of chronic kidney disease in
children: a machine learning–based score. Eur J Pediatr.

[21] Lebel A, Teoh C, Zappitelli M (2020). Long-term complications of acute kidney injury in
children. Curr Opin Pediatr.

[22] Konstantyner T, Sesso R, de Camargo MF, de Santis Feltran L, Koch-Nogueira PC (2015). Pediatric chronic dialysis in Brazil: epidemiology and regional
inequalities. PLoS One.

[23] Rees L, Schaefer F, Schmitt CP, Shroff R, Warady B (2017). Chronic dialysis in children and adolescents: challenges and
outcome. Lancet Child Adolesc Health.

[24] Santos GPA, Sesso R, Lugon, Neves PDMM, Barbosa AMP, Rocha NC (2024). Geographic inequities in hemodialysis access: a call to reassess
dialysis facility locations in Brazil. J Nephrol.

[25] Palma LMP, Penido MGMG, Bresolin NL, Tavares MS, Sylvestre L, Andrade OVB (2022). Pediatric peritoneal dialysis in Brazil: a discussion about
sustainability. A document by the Brazilian Society of Nephrology, the
Brazilian Society of Pediatrics, the Brazilian Association of Organ
Transplantation, and the Brazilian Association of Dialysis and Transplant
Centers. J Bras Nefrol.

[26] Koch-Nogueira PC, Carvalho MFC, Feltran LS, Konstantyner T, Sesso R (2016). Inequality in pediatric kidney transplantation in
Brazil. Pediatr Nephrol.

[27] Raina R, Sethi SK (2022). Pediatric to adult transition: identifying important
comorbidities and considerations for adult and pediartric nephrology health
care teams. Adv Chronic Kidney Dis.

