# Supplementary material for: Standard of care for children and adolescents with chronic kidney disease and its Peculiarities: a call to action!
Source: J Bras Nefrol. 2025 Dec 19;48(1):e20250178. doi: 10.1590/2175-8239-JBN-2025-0178en (PMC12716762; doi:10.1590/2175-8239-JBN-2025-0178en)
Supplement: Translation

# Translation: Linha de cuidado da criança e do adolescente com doença renal crônica e suas Peculiaridades: um chamado à ação!

**DOI:** 10.1590/2175-8239-jbn-2025-0178pt

**Authors:** Lilian Monteiro Pereira Palma, Maria Goretti Moreira Guimarães Penido, Emília Maria Dantas Soeiro, Marcelo de Sousa Tavares, Clotilde Druck Garcia, Arnauld Kaufman, Suzana Aparecida Greggi de Alcantara, Olberes Vitor Braga de Andrade, Káthia Liliane da Cunha Ribeiro Zuntini, Roberta Mendes Lima Sobral, Vandrea Carla de Souza, Rejane de Paula Bernardes, Maria Cristina de Andrade, Nilzete Liberato Bresolin, Paulo Cesar Koch Nogueira, Vera Hermina Kalika Koch

**Affiliations:** 1 Universidade Estadual de Campinas, Hospital de Clínicas, Departamento de Pediatria, Nefrologia Pediátrica, Campinas, SP, Brasil.; 2 Santa Casa de Belo Horizonte, Unidade de Nefrologia Pediátrica, Belo Horizonte, MG, Brasil.; 3 Universidade Federal de Pernambuco, Área Acadêmica de Pediatria Recife, PE, Brasil.; 4 Instituto de Medicina Integral Professor Fernando Figueira (IMIP), Unidade Renal Pediátrica, Recife, PE, Brasil.; 5 Universidade Federal Ciências da Saúde do Rio Grande do Sul, RS, Brasil.; 6 Santa Casa de Porto Alegre, Transplante Pediátrico, Porto Alegre, RS, Brasil.; 7 Universidade Federal do Rio de Janeiro, Rio de Janeiro, RJ, Brazil.; 8 Universidade de São Paulo, Hospital das Clínicas da Faculdade de Medicina de Ribeirão Preto, Nefrologia Pediatrica, SP, Brasil.; 9 Santa Casa de Misericórdia de São Paulo, Faculdade de Ciências Médicas, Departamento de Pediatria, São Paulo, SP, Brasil.; 10 Hospital Infantil Albert Sabin, Fortaleza, CE, Brasil.; 11 Universidade Federal da Bahia, Hospital Universitário Prof. Edgard Santos, Nefrologia Pediátrica, Salvador, BA, Brasil.; 12 Universidade de Caxias do Sul, Programa de Pós Graduação em Ciências da Saúde, Caxias do Sul, RS, Brasil.; 13 Instituição: Clínica NefroKids, Curitiba, PR, Brasil.; 14 Universidade Federal de São Paulo, Escola Paulista de Medicina, Nefrologia Pediátrica – São Paulo, SP, Brasil.; 15 Universidade Federal de Santa Catarina, Hospital Joana de Gusmão, Nefrologia Pediátrica, Florianópolis, SC, Brasil.; 16 Hospital Samaritano, São Paulo, SP, Brasil.; 17 Universidade de São Paulo, SP, Brasil.

**Keywords:** Padrão de Cuidado, Insuficiência Renal Crônica, Nefrologia Pediátrica, Criança, Adolescente

## Abstract

A Linha de Cuidado em Nefrologia Pediátrica é essencial para o diagnóstico, acompanhamento e manejo de crianças com Doença Renal Crônica (DRC). A detecção precoce de alterações renais é fundamental, uma vez que muitas condições podem ser silenciosas, mas ter grande impacto no crescimento e desenvolvimento da criança. A estrutura da Linha de Cuidado inclui triagem neonatal, avaliação de condições predisponentes à doença renal, cálculo da estimativa da função renal e classificação do estágio da DRC, visando à programação de intervenções terapêuticas medicamentosas e não medicamentosas. Desafios como a escassez de especialistas, a necessidade de exames e o acesso desigual aos serviços reforçam a importância de políticas públicas robustas e programas de treinamento. Protocolos personalizados são recomendados para retardar a progressão para Terapia Renal Substitutiva (TRS). Em setembro de 2024, foi lançado o Protocolo Clínico e Diretrizes Terapêuticas (PCDT) para DRC em adultos. Neste artigo de opinião, um grupo de nefrologistas pediátricos comenta as perspectivas para a criação de um PCDT para crianças e adolescentes com DRC.

## Introdução

No dia 16 de setembro de 2024, foi aprovado o Protocolo Clínico e Diretrizes Terapêuticas (PCDT) das Estratégias para Atenuar a Progressão da Doença Renal Crônica (DRC)^1^ em adultos. O documento considera a prevalência crescente da DRC no Brasil (9% da população)^2^ e seu impactona saúde pública, com os elevados custos de manutenção de pacientes em todas as formas de Terapia Renal Substitutiva (TRS): hemodiálise, diálise peritoneal e transplante renal.

Estudos mostram que a implantação de uma “Linha de Cuidado em Nefrologia” melhora a qualidade de vida dos pacientes com DRC, retarda a progressão da doença renal, bem como a necessidade de diálise e de transplante renal, proporcionando economia de recursos financeiros^3^ e naturais.

O rastreamento da DRC nos grupos de risco é fundamental. Cabe à Atenção Primária (AP) em saúde o reconhecimento de condições associadas à possível progressão da doença renal. Para os adultos, preconiza-se o melhor controle da hipertensão arterial sistêmica (HAS) e do *diabetes mellitus*, a detecção precoce da DRC por meio de fórmulas para calcular a Taxa de Filtração Glomerular (TFG), exame de urina e de imagem, além de orientações para modificação de hábitos de vida. No caso dos adultos, o arsenal terapêutico proposto no PCDT^1^inclui inibidores da enzima de conversão da angiotensina (IECA), bloqueadores de receptores da angiotensina, antagonistas de aldosterona (BRA), inibidores de cotransportador sódio-glicose 2 (iSGLT2) e manejo conservador da anemia, da doença mineral óssea, da HAS, da acidemia e da doença cardiovascular. Entretanto, essas diretrizes não abrangem as necessidades da população pediátrica.

### Quais São as Necessidades da População Pediátrica?

A Nefrologia Pediátrica é a área de atuação da Pediatria responsável por diagnosticar, acompanhar e tratar condições que possam afetar os rins de crianças e adolescentes. As causas mais frequentes de nefropatias nessa faixa etária diferem das observadas na população adulta e compreendem as malformações do trato urinário, glomerulopatias, doenças renais genéticas, tubulopatias e condições adquiridas (lesão renal secundária à sepse e a outras doenças infecciosas, uso de drogas e quimioterápicos nefrotóxicos). Desde a concepção, um ambiente intrauterino desfavorável pode promover adaptações (fenômenos epigenéticos) que comprometem a maturação renal^4^. Esse processo é denominado Programação Fetal e Pós-natal das doenças renais do adulto.

Os avanços tecnológicos permitiram a sobrevida de bebês prematuros, de recém-nascidos com baixo peso^5^, de crianças submetidas a cirurgias complexas, de crianças com câncer que receberam quimioterápicos nefrotóxicos e daquelas submetidas a internações em Unidades de Terapia Intensiva. Sob essas condições, há potencial para perda de função renal lenta e gradual ao longo de anos, e o reconhecimento e a intervenção iniciais recaem sobre a equipe de saúde da AP.

Outro fator importante é o aumento da obesidade pediátrica. Como consequência, a HAS vem se tornando cada vez mais prevalente nessa população e associa-se à piora da função renal quando não tratada. Pesquisas mostram que programas públicos de intervenção contra a obesidade infantil nas escolas não são eficientes se não considerarem outras fontes de apoio a um estilo de vida saudável, principalmente vindas da família^6^ (alimentação saudável, combate ao sedentarismo, estímulo à atividade física e higiene de sono). Para obter sucesso, é necessária uma abordagem integral do paciente e de sua família. Uma abordagem exclusivamente médica não é suficiente para alcançar uma terapêutica mais efetiva, não somente na AP, mas principalmente na Secundária e na Terciária. Esses dados justificam a criação de uma Linha de Cuidado em Nefrologia Pediátrica (Figura 1).

**Figura 1:** Esquema geral da Linha de Cuidado em Nefrologia Pediátrica.

### Como Fazer a Prevenção da DRC em Crianças?

#### Cuidando da Futura mãe:

- Evitar o uso de IECA, BRA, anti-inflamatórios não esteroides (AINEs), drogas ilícitas, fumo e álcool
- Prevenir e tratar o sobrepeso/obesidade, a síndrome metabólica e o diabetes gestacional
- Diagnosticar e tratar dislipidemias
- Diagnosticar e tratar a HAS
- Controlar doenças infecciosas (rubéola, toxoplasmose, citomegalovírus etc.)
- Realizar aconselhamento genético
- Estimular o uso de ácido fólico
- Cuidar da nutrição dessa futura mãe
- Reconhecer e diagnosticar doenças genéticas com potencial de transmissão ao feto
- Garantir assistência pré-natal adequada, com ultrassonografia fetal de qualidade, para identificação de malformações do trato urinário e disrafismos espinhais
- Encaminhar gestantes para hospitais de assistência terciária (neonatologia e nefrologia pediátrica) quando diagnosticada malformação do trato urinário

Para atingir esses objetivos, é necessária uma ação junto aos ginecologistas e obstetras, bem como uma ação primária com a equipe multi-profissional das Unidades Básicas de Saúde (UBSs).

#### Cuidando do Recém-Nascido (RN)

- Evitar o uso de IECA, BRA, AINEs, além de uso pela genitora de drogas ilícitas, fumo e álcool
- Implementar medidas educativas junto à equipe de neonatologia para o acompanhamento de RNs com risco de progressão da DRC
- Ressaltar que a injúria renal aguda (IRA) é um importante fator de risco de progressão para a DRC (esses bebês necessitam de acompanhamento após a alta, com avaliação da função renal, da presença de proteinúria/microalbuminúria e controle dos níveis de pressão arterial)
- Minimizar a exposição a agentes nefrotóxicos (aminoglicosídeos, anti-inflamatórios não hormonais)
- Destacar a importância da creatinina sérica e disseminar tabelas com valores de referência por faixa etária, além da fórmula para o cálculo da TFG

#### Cuidando do lactente

- Evitar o uso de IECA (principalmente antes das 44 semanas de idade gestacional corrigida), BRA, AINEs, além de uso pela genitora de drogas ilícitas, fumo e álcool
- Incentivar o aleitamento materno
- Aferir a pressão arterial nos grupos de risco
- Garantir a nutrição adequada do lactente
- Avaliar o *catch-up* (recuperação) do crescimento pós-natal
- Encaminhar os pacientes com IRA para acompanhamento com o nefrologista pediátrico
- Esclarecer sobre a importância da creatinina sérica e disseminar tabelas com valores de referência por faixa etária, além da fórmula para cálculo da TFG

Para atingir esses objetivos, é necessária uma ação conjunta entre pediatras, pais/cuidadores e equipe multiprofissional das UBSs

#### Cuidando da Criança e do adolescente

- Orientar a introdução alimentar
- Promover a educação alimentar
- Aferir, anualmente, a pressão arterial de crianças eutróficas, a partir dos três anos de idade
- Aferir frequentemente a pressão arterial em pacientes com menos de três anos de idade nos grupos de risco
- Incentivar a prática de atividade física sob supervisão profissional
- Prevenir sobrepeso/obesidade e dislipidemias
- Orientar sobre os malefícios do uso de drogas ilícitas, fumo e álcool
- Esclarecer sobre a importância da creatinina sérica e disseminar tabelas com valores de referência por faixa etária, além da fórmula para cálculo da TFG
- Em todas as idades, orientar a avaliação de dados antropométricos, a mensuração da creatinina sérica para cálculo da TFG e a aferição da pressão arterial, uma vez que essas medidas são sinais de alerta para doenças crônicas. Além disso, orientar sobre a importância da vacinação adequada em todas as faixas etárias

Ressalta-se o papel fundamental da equipe de enfermagem, dos nutricionistas, dos assistentes sociais e da equipe de saúde mental no apoio psicológico e emocional de todas as linhas envolvidas nesse cuidado.

### Quais São as Peculiaridades do Diagnóstico das Doenças Renais na População Pediátrica? Quais as Diferenças em Relação Aos Adultos?

O diagnóstico da DRC pediátrica inicia-se com a avaliação da história familiar e perinatal da criança, complementada pela ultrassonografia fetal, um instrumento diagnóstico fundamental para a detecção de malformações do sistema urinário, bem como de manifestações morfológicas de doenças geneticamente determinadas.

A doença renal é frequentemente assintomática; entretanto, pode se apresentar com sinais e sintomas, tais como alterações na urinálise, infecção do trato urinário, distúrbios hidroeletrolíticos ou ácido-base, anormalidades congênitas do rim e do trato urinário, envolvimento renal em doença sistêmica, doença glomerular, doença tubular renal e HAS. Portanto, a anamnese completa e o exame físico são fundamentais para o diagnóstico. O exame físico deve obrigatoriamente incluir a aferição da pressão arterial, e os antecedentes familiares e pessoais devem ser sempre investigados. As doenças renais pediátricas e as do adulto diferem quanto às causas, à apresentação e às consequências em longo prazo (efeitos renais evolutivos, crescimento e desenvolvimento neurocognitivo). É fundamental reconhecer que, embora os princípios básicos de diagnóstico e tratamento sejam semelhantes, o impacto da doença renal sobre o crescimento e o desenvolvimento requer considerações específicas para cada faixa etária da criança e do adolescente^7^.

### Desafios para Estimar a TFG na População Pediátrica

A dificuldade em definir a DRC pediátrica utilizando a TFG é demonstrada pela definição do KDIGO (*Kidney Disease Improving Global Outcomes*) que, em sua última edição^8,9^, estabelece o limite inferior da normalidade para a TFG estimada (TFGe) – calculada a partir da dosagem de creatinina sérica – como sendo ≥90 mL/min/1,73 m^2^ para crianças e adolescentes com idade >2 anos. Entre 1 e 2 anos de idade, a TFG da criança já atinge níveis comparáveis aos do adulto.

### Como a TFG Varia em Crianças Abaixo de 2 Anos de Idade?

A nefrogênese se completa entre a 32^a^ e a 36^a^semana de gestação. Estima-se que, para cada quilograma adicional de peso, formem-se aproximadamente 250.000 néfrons por rim. Ao final da gestação, esse processo culmina com cerca de um milhão de néfrons em cada rim de um recém-nascido a termo^13^. Nos neonatos a termo, não ocorre nefrogênese pós-natal. Em prematuros, porém, o processo pode persistir até cerca de 40 dias de vida, geralmente cessando duas semanas antes da idade corrigida esperada. Nesse ambiente extrauterino, os néfrons formados são mais vulneráveis a malformações e disfunções, reforçando a importância de estratégias de proteção renal no período neonatal^10,11,12^. Uma porção significativa dos néfrons se forma no 3º trimestre da gestação (60%); portanto, quanto mais extrema a prematuridade, menor o número total de néfrons ao nascimento^14^. A TFG do neonato correlacionase com a idade gestacional ao nascimento e é menor no prematuro do que no RN a termo. Nos primeiros dias de vida, o neonato apresenta níveis séricos de creatinina próximos aos maternos, passando, em seguida, a mostrar incremento progressivo da TFG, de acordo com o número de néfrons funcionantes e a massa muscular^15^ (Tabela 1).

**Tabela 1:** Taxa de Filtração glomerular (depuração de inulina) na população pediátrica com idade < 2 anos^16^

Idade	TFG média ± DP mL/min/1.73 m^2^ SC
RN prematuro 1–7 dias	18,7 ± 5,5
RN prematuro com 1,5–4 meses	67,4 ± 16,6
RN termo 1–3 dias	20,8 ± 5,0
RN termo 1–3 meses	85,3 ± 35,1
RN termo 7–12 meses	96,2 ± 12,2
RN termo 1–2 anos	105,2 ± 17,3

Abreviaturas – TGF: taxa de filtração glomerular; DP: desvio padrão; SC: superfície corporal; RN: recém-nascido.

A avaliação da TFG em crianças a partir de um ano de idade e em adolescentes ocorre por meio de fórmulas e é denominada TFG estimada (TFGe)^17^. A equação sugerida para essa avaliação é a CKiDU-25, com base na creatinina sérica ou plasmática, ou na cistatina C^18^, e que pode ser calculada de forma online na página **(https://ckid-gfrcalculator.shinyapps.io/eGFR/**). O KDIGO 2024^9^ recomenda que o cálculo da TFGe de crianças e adultos jovens com alterações de massa muscular seja determinado com base na cistatina C (por exemplo, em casos de malformações e atrofia de membros inferiores, disrafismos de coluna e doenças musculares).

### Quando Começa o Tratamento Conservador da DRC?

O tratamento conservador deve ser iniciado após o diagnóstico da DRC, ainda nos estágios iniciais, a fim de aumentar as chances de preservação da função renal e retardar a necessidade de TRS. Alguns sinais e sintomas alertam para a probabilidade da doença, contribuindo para o diagnóstico precoce^19,20^. São eles:

1. Sinais relacionados a malformações do trato urinário (alterações urológicas na ultrassonografia antenatal, ITU recorrente, jato urinário anormal);
2. Sinais de doenças glomerulares (história de HAS, edema de face e corpo, presença de espuma na urina);
3. Parada (achatamento) na curva de crescimento;
4. Sinais de perda da capacidade de concentração da urina (poliúria, polidipsia, noctúria);
5. Baixo peso ao nascimento;
6. História de internação no período neonatal^20^(especialmente quando a mãe recebe alta hospitalar, mas o RN permanece internado).

#### Pilares do tratamento conservador

- Preservação da função renal por meio do controle frequente da TFG, manejo da hidratação e dos distúrbios minerais, evitandose nefrotoxinas
- Vigilância do crescimento e tratamento da baixa estatura
- Modificação do estilo de vida: prática de atividade física adequada para a idade, dieta saudável e redução da ingestão de sódio, evitando-se restrição proteica pelo risco de comprometimento do crescimento
- Manejo de sintomas (náusea, falta de apetite e sentimentos relacionados à doença)
- Detecção precoce de albuminúria e proteinúria, com controle por meio de medidas medicamentosas e não medicamentosas
- Gerenciamento de HAS, anemia, distúrbio mineral ósseo e outras comorbidades
- Manutenção da qualidade de vida (absenteísmo escolar com grande prejuízo à formação cognitiva e social)
- Incentivo à participação das famílias e dos pacientes nas tomadas de decisão sobre todos os aspectos do tratamento das crianças (Figura 2).

**Figure 2:** Pilares do tratamento conservador da doença renal crônica na população pediátrica.

### TRS – Diálise e Transplante Renal – Quais São as Necessidades?

Em 2015, um estudo mostrou que, no Brasil, o número de crianças em diálise ou transplantadas chegava a 1.283, com uma prevalência de 20 casos por milhão de população (pmp) e incidência de 6,6 pmp. Verificou-se disparidade entre as regiões do país, tanto em relação ao perfil da população, quanto ao tipo e à qualidade do tratamento ofertado^21^.

A diálise peritoneal (DP) é uma modalidade de suporte renal artificial de alta qualidade, baixo custo e considerada a terapia de escolha para crianças e adolescentes. Seus resultados clínicos, bem como aqueles relacionados à qualidade de vida, são comparáveis – se não superiores – aos obtidos com a hemodiálise (HD). Mais de 50% dos pacientes pediátricos com DRC em estágio final iniciam a TRS por meio da DP. O objetivo dessa terapia é preparar o paciente para um transplante renal oportuno.

As vantagens da DP, quando comparada à HD, incluem: preservação da função renal residual, menos restrições dietéticas, possibilidade de frequência escolar, ausência de necessidade de acesso vascular, menor risco de perda potencial do crescimento, preservação dos acessos vasculares e manutenção do convívio social. Diversos estudos apontam essa terapia como a melhor opção para bebês e crianças pequenas^22^.

A falta de oferta de DP para pacientes pediátricos inviabiliza a continuidade da vida de muitos que não possuem o peso mínimo necessário para a realização de HD. Recém-nascidos e lactentes com poucos meses de idade, bem como aqueles com baixo peso, prematuros extremos ou que nascem com problemas, têm na DP sua única chance de sobrevivência até um eventual transplante renal.

Um estudo brasileiro publicado em 2024 demonstrou a desigualdade regional na distância de deslocamento até as unidades de diálise no país^23^. No Sul e Sudeste, onde há uma densidade maior de unidades, os pacientes contam com maior disponibilidade regional de centros, percorrendo distâncias mais curtas (mediana de 27,6 km até a unidade de diálise). Em contraste, na região Norte, essa distância é significativamente maior (mediana de 84,3 km), e 77% dos pacientes precisam viajar mais de 40 km para realizar o tratamento. Em 19 de fevereiro de 2024, a Agência Câmara de Notícias anunciou um projeto que estabelece que as unidades de hemodiálise devem estar localizadas a menos de 100 km da moradia do paciente. Trata-se do Projeto de Lei nº 6133/23 (camara.leg.br), que obriga a administração pública a assegurar a existência de unidades de HD em um raio de até 100 km dos pacientes. O texto também prevê mecanismos de fiscalização e monitoramento para garantir o cumprimento da limitação de distância e a qualidade do atendimento nas unidades de HD. Até o momento da publicação deste artigo, o projeto ainda aguardava análise pelas comissões de Saúde, Finanças e de Constituição e Justiça da Câmara dos Deputados.

A comunidade de Nefrologia Pediátrica brasileira tem crescido e já está presente em todos os estados brasileiros e no Distrito Federal. Todavia, o número de serviços que contam com nefrologistas pediátricos ainda é insuficiente para suprir a demanda de pacientes que necessitam de TRS (dados do Departamento de Nefrologia Pediátrica da Sociedade Brasileira de Nefrologia). Embora os nefrologistas pediátricos sejam treinados e capacitados para atender as mais diversas doenças renais e suas complicações, nem todos os serviços dispõem do suporte necessário para iniciar diálise em crianças e recém-nascidos. Existe uma discrepância regional significativa e, em algumas situações, os pacientes não conseguem aguardar a transferência para um centro capacitado para o tratamento dialítico, resultando em óbito no serviço de origem.

Em 2022, foi realizado um estudo transversal brasileiro com o objetivo de identificar o número de centros de TRS no país que utilizam a DP em pediatria^24^. O estudo mostrou que 212 pacientes pediátricos estavam em programa de DP em 37 centros, sendo a maioria composta por crianças de 0 a 12 anos. A DP automatizada foi a principal modalidade utilizada (86%), e em 74% dos casos a fonte pagadora foi o Sistema Único de Saúde. Neste estudo, a Vantive^®^ (anteriormente Baxter^®^) foi identificada como a principal empresa fornecedora de máquinas e insumos para a DP pediátrica no Brasil, detendo mais de 80% do mercado. Contudo, cerca de 25% dos serviços relataram enfrentar desabastecimento de insumos nos meses anteriores, resultando na conversão de pacientes pediátricos de DP para HD em 19 casos (51%).

Para a grande maioria das crianças com DRC, o tratamento de escolha é o transplante renal. No entanto, um mapeamento realizado em 2015 revelou uma distribuição desigual na frequência de transplantes entre as regiões do Brasil^25^. A menor taxa foi registrada no Centro-Oeste, com 0,4 casos por milhão de população relacionada à idade (pmp), enquanto a maior foi observada na região Sul, com 8,3 casos pmp. Crianças das regiões Norte e Centro-Oeste apresentaram uma probabilidade 3–4 vezes menor de receber um transplante de doador falecido (p < 0,05). Além da região geográfica, a idade dos receptores e o índice de desenvolvimento local também influenciaram os resultados. A probabilidade de receber um transplante renal foi muito baixa entre crianças pequenas nas regiões Norte e Centro-Oeste. Os autores recomendam o encaminhamento dessas crianças para um Centro de Transplante Renal quando a TFG estiver <30mL/min/1,73m^2^, o que permite avaliação e seguimento cuidadosos, além do preparo do trato urinário inferior, quando necessário, visando aumentar a possibilidade de realização de transplante renal preemptivo.

### Que Estratégias São Necessárias para Estabelecer uma Linha de Cuidado em Pediatria?

Diferentemente do que ocorre em adultos, ainda não existe uma sistematização da Linha de Cuidado em Nefrologia Pediátrica. O fato de a maioria dos estudos clínicos excluir pacientes pediátricos, sobretudo aqueles com idade inferior a 12 anos, faz com que o uso de grande parte das medicações seja *off label*. Além disso, a menor ingestão alimentar e as restrições dietéticas nas fases mais avançadas da DRC prejudicam o crescimento e o desenvolvimento da criança. Os requisitos atualmente utilizados pelas farmácias de alto custo para a dispensação de medicamentos não contemplam as necessidades pediátricas. Essas questões, por si só, já justificam um algoritmo próprio para os cuidados na DRC pediátrica, envolvendo uma equipe multiprofissional composta por psicólogos, nutricionistas, enfermeiros e terapeutas ocupacionais.

- Desenvolver uma diretriz bipartite, com membros dos Departamentos de Nefrologia Pediátrica das Sociedades Brasileiras de Nefrologia e de Pediatria, com a criação de um algoritmo específico para a pediatria, semelhante ao PCDT para adultos, publicado em 16 de setembro de 2024^1^;
- Incluir o tratamento medicamentoso e a promoção de hábitos de vida saudáveis, tendo em mente que o pilar primordial é o apoio e o interesse da família;
- Fornecer orientações nutricionais em DRC, com opções práticas e de baixo custo de cardápios adequados para cada estágio da doença, de acordo com a faixa etária;
- Estudar a liberação de medicamentos com custo-efetividade comprovada por estudos clínicos e já aprovados em mercados internacionais para uso em adultos, seguindo as diretrizes regulatórias. Exemplos: sevelâmer, cinacalcete, inibidores de SGLT2 e ciclossilicato de zircônio;
- Enquanto se aguarda a aprovação para uso pediátrico de determinados medicamentos, considerar o uso *off label* daqueles com eficácia e custo-efetividade comprovadas na população adulta, como os inibidores de SLGT2 (gliflozinas);
- Tornar realidade a criação de equipes multi-profissionais voltadas para uma abordagem integral das crianças com DRC e de suas famílias;
- Referenciar precocemente os pacientes com DRC para Centros Transplantadores Pediátricos;
- Planejar a Transição de Cuidado para a Nefrologia de adultos com base em iniciativas que fomentem a autonomia^26,27^.

## Conclusão

Definir uma Linha de Cuidado em Nefrologia Pediátrica e envolver a família no tratamento são passos fundamentais para retardar o início da TRS. Promover o acesso rápido aos centros transplantadores renais e capacitar novos serviços é uma urgência nacional.

Vamos à ação!!

## Data Availability

Nenhum dado novo foi gerado ou analisado neste estudo.
